# Recto-vaginal septum cystadenocarcinoma: a case report and review of the literature

**DOI:** 10.1186/s12905-016-0300-z

**Published:** 2016-05-03

**Authors:** Sophie Warembourg, Mélanie Cayrac, Gauthier Rathat, Arash Rafii

**Affiliations:** Department of Gynecology, Nîmes Hospital, Montpellier-Nîmes University, 4 rue Professeur Robert Debré, 30029 Nimes, France; Department of Gynecology, Montpellier Hospital, Montpellier-Nîmes University, 371 avenue du Doyen Gaston Giraud, 34090 Montpellier, France; Department of Genetic Medicine, Weill Cornell Medical College, New York, NY USA

**Keywords:** Serous adenocarcinoma, Recto-vaginal septum, Primary peritoneal carcinoma

## Abstract

**Background:**

Carcinoma of the recto-vaginal septum is a quite rare location and related to peritoneal and primary ovarian carcinomas. There are only few reports in the literature with a very poor prognosis.

**Case presentation:**

Here we report the case of a 63 years old woman with past medical history of left oophorectomy presenting with a pelvic pain. The magnetic resonance imaging (MRI) demonstrated a 10 cm mass located in the recto-vaginal septum. A block resection was performed allowing the retrieval of a 10 cm solid tumor of the recto-vaginal septum. Peritoneal biopsies and the right ovary were normal the final diagnosis was cystadenocarcinoma of the recto-vaginal septum. The patient received adjuvant chemotherapy and displays no sign of recurrence 36 months after diagnosis.

**Conclusion:**

The management of recto-vaginal septum carcinoma with en bloc resection should be performed to avoid peritoneal spread and improve prognosis.

## Background

Occurrence of a carcinoma of a recto vaginal septum is an exceptional presentation. Few cases of such disease have been described in the literature with a poor prognosis due to local early recurrence [1, 2]. While the management of the disease is similar to an ovarian carcinoma in particular for adjuvant treatment, the specific prognosis of such lesion remains unknown. Moreover surgical management of recto-vaginal septum carcinoma can be a challenge as specific techniques might be required to reduce the risk of peritoneal spread of the tumor. Here we report a case of a recto vaginal septum with emphasis on the surgical procedure, as radicality and a bloc resection might be important factors for local control.

## Case presentation

A 63 year old patient presented to the clinic for pelvic pain. Her past medical history included a hypothyroidy and dyslipidemia. The BMI of the patient was normal (BMI 19,1). Her family history was notable for an aunt (father side) presenting with an ovarian carcinoma.

Interestingly the patient had an oophorectomy performed 4 years before for a 6 cm left ovarian cyst by laparoscopy. During the previous surgery the cyst was adherent to the pelvic peritoneum and fallopian tube. There was an exophytic part adhering tightly to the rectum and Douglas Pouch. Rectal examination could not find any rectal or recto-vaginal nodule. After adhesiolysis a left adnexectomy was performed without particular difficulties. The pathology examination was consistent with a benign serous cystadenoma of the ovary. The left fallopian tube was normal. She underwent a CT scanner and a colonoscopy after the surgery which with no particular findings. She underwent regular gynecologic examination as well as yearly pelvic ultrasound.

**Fig. 1:**
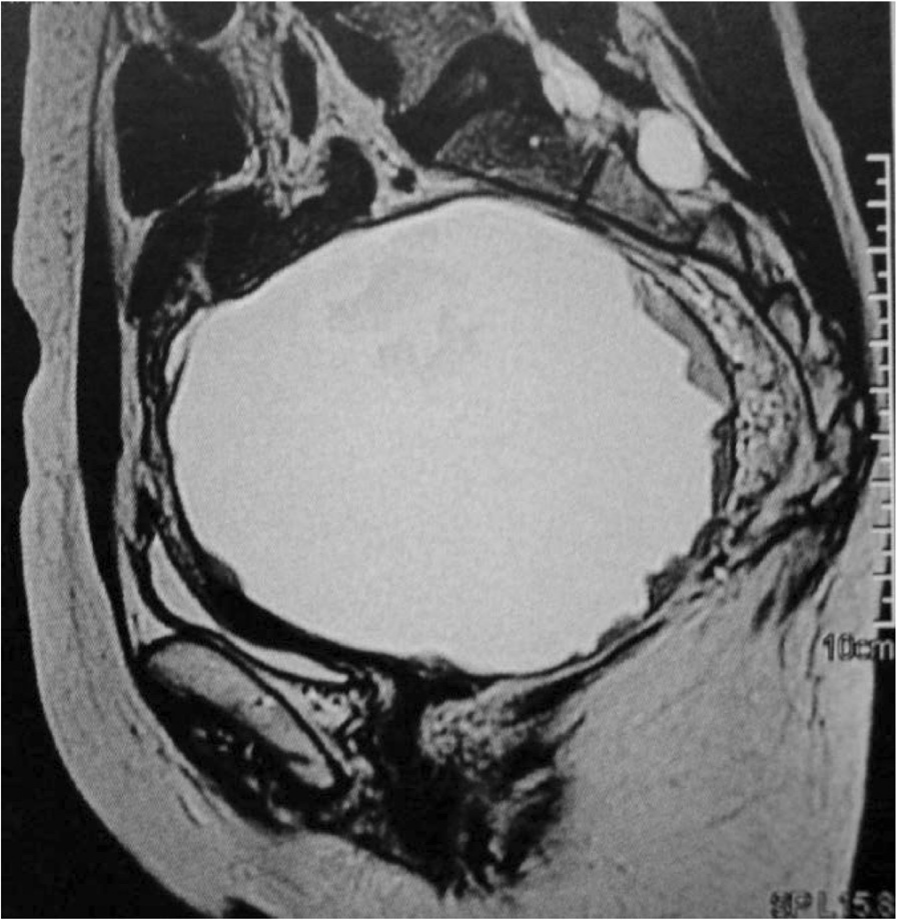
Sagittal T2 weighted pelvic MRI images

**Fig. 2:**
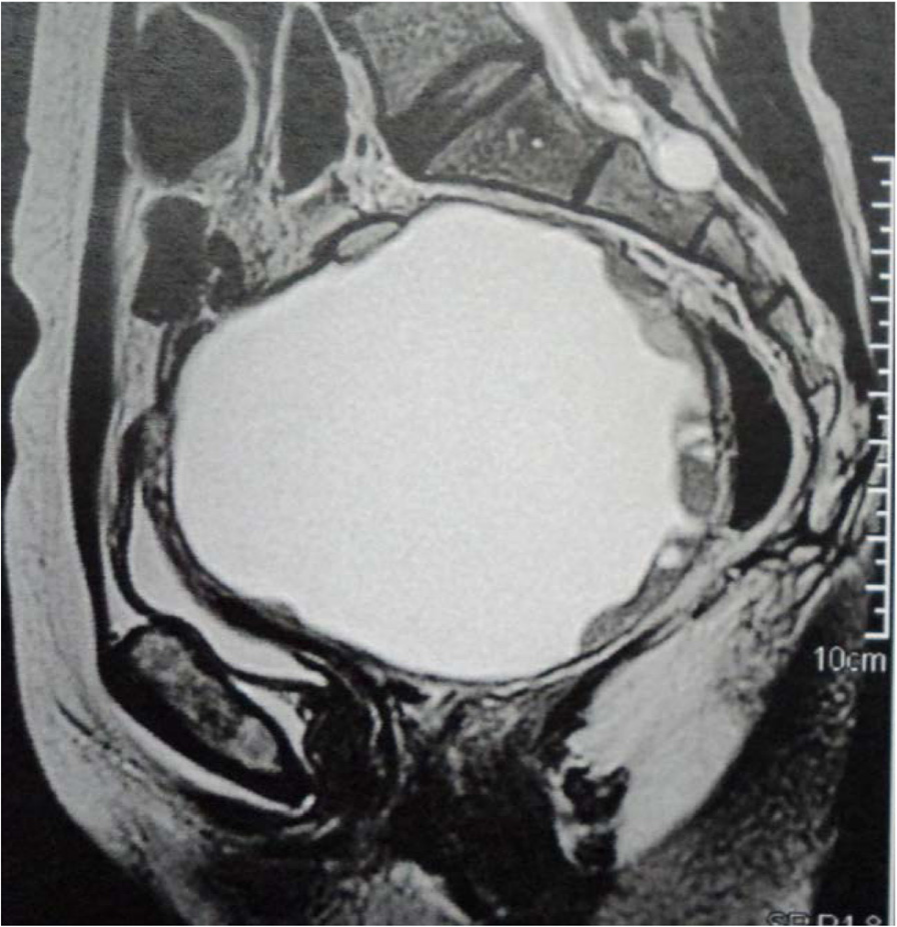
Sagittal T2 weighted pelvic MRI images

Four years later the pelvic ultrasound demonstrated a retro-uterine solid 11 cm cyst. Clinical examination found an immobile pelvic mass pushing the vagina anteriorly. There was no sign of vaginal or rectal invasion. Concordantly a pelvic MRI [Figs. 1 and 2] confirmed a 10*12 cm cystic structure with thick walls. The content of the cyst looked heterogeneous and the vegetations within the cyst were uptaking gadolinium. There was no sign of rectal invasion, but minimal fluid in the abdomen and a single external iliac node looked suspicious. CA125 was above normal (112 UI/L).

**Fig. 3:**
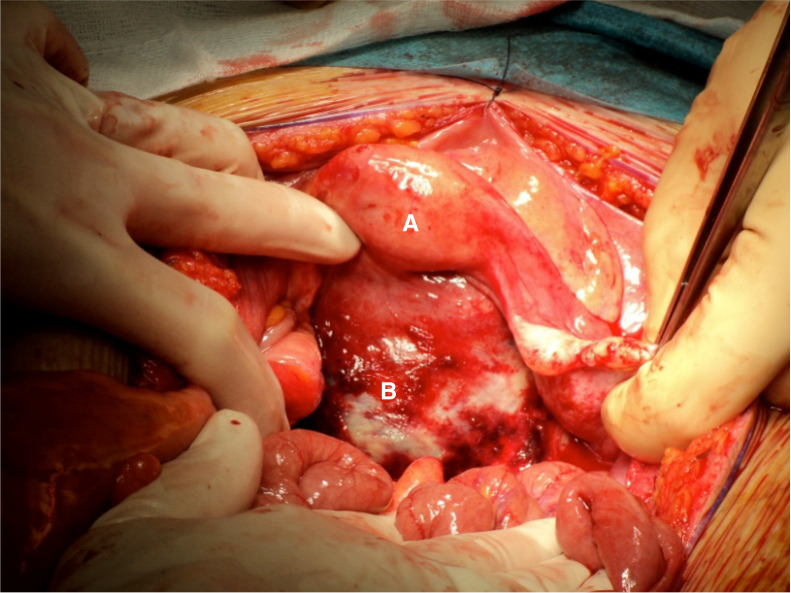
Per-operative view of tumor located on recto-vaginal septum. *a* Uterus. *b* Recto-vaginal septum including the tumor mass

**Fig. 4:**
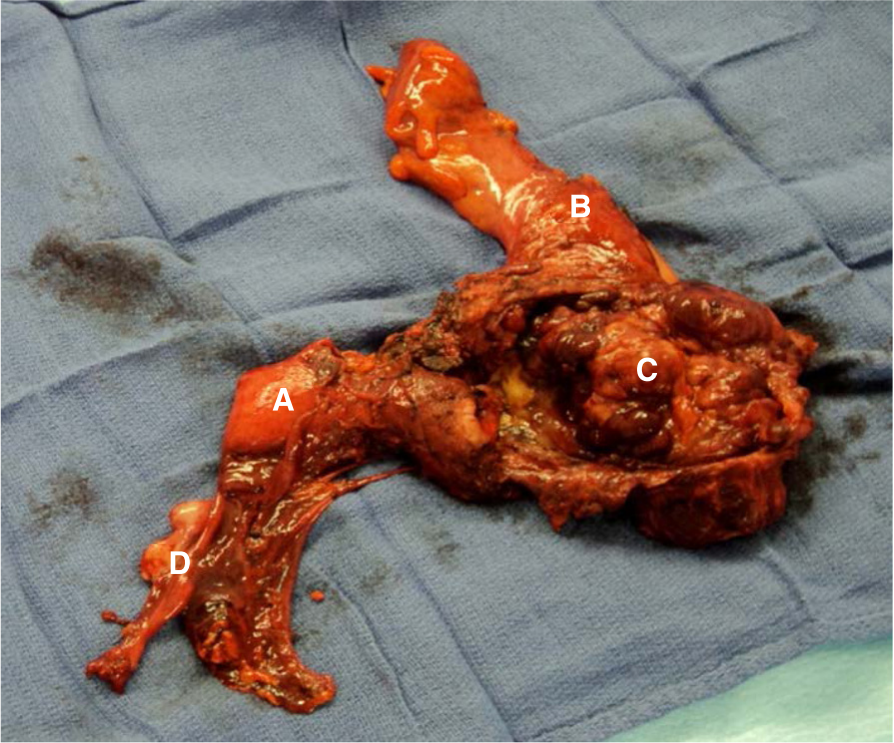
« en-bloc » resection. *a* Uterus. *b* Rectum. *c* Tumoral mass. *d* Right ovary

**Fig. 5:**
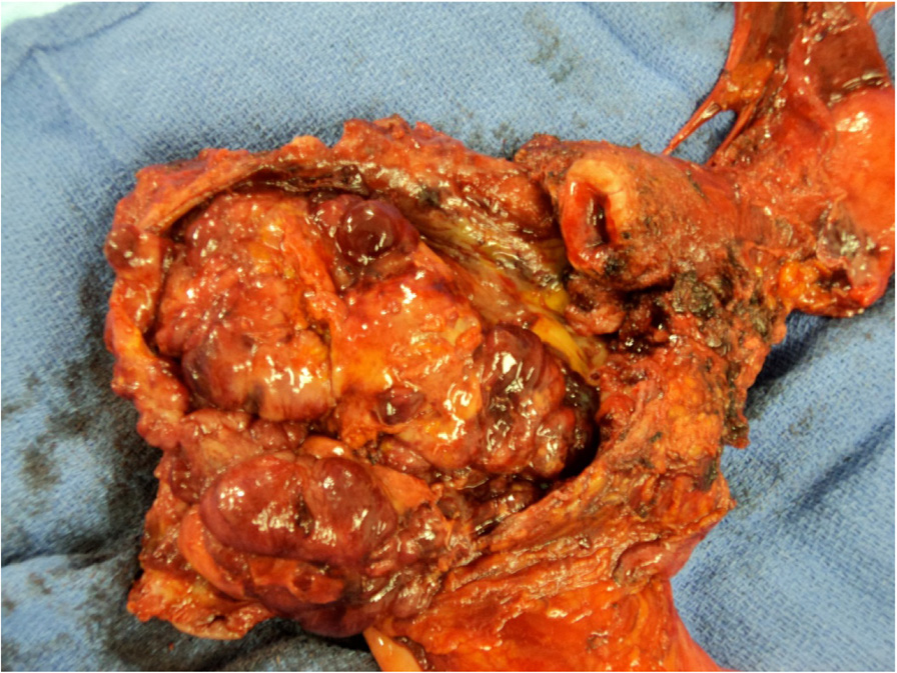
Recto-vaginal septum tumor

A midline laparotomy was performed. On abdominal entry the mass seemed enclaved in the recto-vaginal septum [Fig. 3]. The right adnexa was normal. Several enlarged lymph nodes were perceived with the bigger located on the right iliac bifurcation. A frozen section of this node confirmed a metastatic involvement with a poorly differentiated carcinoma. The patient underwent an « en bloc » resection through a retroperitoneal dissection [Figs. 4 and 5].

She underwent a hysterectomy, right adnexectomy, a coloprotectomy with a low colo-rectal anastomosis and protective ileostomy, omentectomy and peritoneal biopsies. The vagina could be preserved. Pelvic and para-aortic lymphadenectomy was performed. Postoperative course was unremarkable. Pathology exam revealed an adenocarcinoma of the recto-vaginal septum, grade 3 consistent with a serous ovarian carcinoma or a primary peritoneal carcinoma (morphological and staining characteristics). A second para-aortic node was metastatic. The right adnexa was normal.

The patient received 6 cycles of carboplatinum and taxol and 21 cycles of bezacizumab (AVASTIN®).

On follow-up the patient is disease free (clinical, imaging and biological assessment) at 36 months after diagnosis.

## Discussion

Serous carcinoma of the recto-vaginal septum is an exceptional presentation presenting multiple clinical challenges. Extra-ovarian Primary Peritoneal Carcinoma (EOPPC) with no ovarian lesion is a described entity [3]. It displays common traits with ovarian serous carcinomas both on clinical presentation and pathological features [4–6]. The main theory for the etiology of this particular disease is the presence of persisting embryologic germinal cells (mullerian duct remnants) able to give rise to primary peritoneal adenocarcinoma [7]. It often presents as a serous carcinoma. A single case has been described in a patient with previous hysterectomy and bilateral salpingo-oophorectomy [8]. A case of recto-vaginal serous carcinoma was reported by Leteurte et al. [1]. The authors described the presence of tumoral foci on the ovary without invasion suggesting a peritoneal primary lesion of the recto-vaginal septum. In this study peritoneal and lymph node involvement was present at diagnosis. The patient recurred after 6 months despite aggressive surgery and adjuvant chemotherapy. Another case of a 2.5 cm tumor was described [2] treated by radical hysterectomy, bilateral salpingo-oophorectomy, lymph node resection and adjuvant radiotherapy. The patient however presented local and regional metastatic spread and died 30 months after diagnosis.

Among the differential diagnosis few patients had a mesothelioma which can be easily be identified on morphological and histological findings. These cases are however rare.

In our case we could not differentiate between primary peritoneal versus primary ovarian tumor. However, the most likely hypothesis is the peritoneum and recto-vaginal septum dissemination of a left ovarian neoplasic foci resected previously. To reduce this risk, in postmenopausic women with a solid and cystic tumor adhered to the rectum, hysterectomy and double salpingo-ophorectomy might be better than simple left adnexectomy. The treatment of primary peritoneal carcinoma is similar to primary ovarian carcinoma with debulking surgery aiming at no residual disease associated to platinium-based chemotherapy. Our surgical approach of « en bloc » resection seems optimal. Indeed using the retroperitoneal sheath for all dissection leads to resection of such tumor without entry in the tumoral mass. Preserving the integrity of the tumor during surgery is mandatory to reduce local dissemination, residual disease and eventually local and distant recurrences.

Most studies report a poor prognosis for primary peritoneal carcinoma related to extensive loco-regional disease with an overall survival around 21 months [6, 9]. In our case, the tumor was limited to recto-vaginal wall and there were no peritoneal lesions. This could impact prognosis, compare to a classical ovarian cancer where the peritoneal extension is the major factor for tumoral dissemination and disease recurrence.

## Conclusion

Cystadenocarcinoma of the recto-vaginal septum is a rare entity. The surgery should emphasize a retroperitoneal approach with an en-bloc resection to avoid as much as possible tumor rupture. Staging and adjuvant treatment should be performed as in primary ovarian carcinosis. The description of more cases would lead to a better understanding of the specificity of such location regarding diagnosis, management and prognosis.

### Consent

Written informed consent was obtained from the patient for publication of this Case report and any accompanying images.

## Abbreviations

CT: computed tomography
MRI: magnetic resonance imaging
